# The relationship between trust and humility: a systematic review

**DOI:** 10.3389/fpsyg.2026.1812466

**Published:** 2026-07-31

**Authors:** Jordon E. Swain, Archie L. Bates, Kate Conkey, Yasmine L. Konheim-Kalkstein

**Affiliations:** United States Military Academy, West Point, NY, United States

**Keywords:** humble, humility, leadership, systematic review, trust

## Abstract

**Purpose:**

This work synthesizes the growing body of research exploring the relationship between the important constructs of humility and trust and addresses four important research questions.

**Design/methodology/approach:**

This systematic review follows the approach prescribed by the updated 2020 Preferred Reporting Items for Systematic Reviews and Meta-Analyses (PRISMA 2020) statement. This review includes so-called “grey literature” to reduce bias and increase its utility.

**Findings:**

Of 76 full text records identified, 56 were deemed eligible and reviewed. Findings reveal that humility, while defined differently by those in different disciplines, engenders trust, but the relationship is complicated. Various mediators and moderators exist that impact the relationship. Further, the relationship between humility and trust may not be one way. Some research suggests that trust can influence humility. There are also numerous theories used to explain—and methods employed to understand—this complex relationship.

**Research limitations/implications:**

Future work can address measurement and methodological shortfalls, as well as explore inconsistencies in results by replicating select studies or examining potential moderators and boundary conditions. Future research could also expand its focus to include foreign language papers to reduce publication bias.

**Originality/value:**

This is the first review to systematically integrate the literature exploring the relationship between the two increasingly important topics of humility and trust. We identify trends and gaps in the existing literature and suggest future work that could better elucidate the relationship between humility and trust.

## Introduction

Trust has long been recognized as essential for initiating, maintaining, and improving relationships in the workplace (Dirks and de Jong, 2022). As a result, it is one of the most frequently explored topics in organizational literature (Burke et al., 2007). Research shows that trust in leadership influences employees’ job satisfaction, commitment to the organization, and retention, as well as their creativity, knowledge sharing, and organizational citizenship behaviors (Dirks and de Jong, 2022), whereas a lack of trust leads to negative outcomes such as reduced innovation, lower engagement, decreased productivity, and higher rates of burnout (Zak, 2017). Despite its known importance, there exists a trust deficit leaders must contend with. A 2023 Gallup Poll found that only 21% of U. S. employees strongly trust their leaders (Herbst, 2025). Further, while 86% of business executives believe employee trust is high in the organizations they lead, only 67% of employees report actually having trust in their employer (PricewaterhouseCoopers, 2024).

While not as widely studied as trust, humility has emerged as a significant topic in leadership and organizational research. Humility has been shown to facilitate well-being, motivation, creativity, innovation, and reduced turnover intention (Chandler et al., 2023). It has also been linked to both improved team performance (Owens and Hekman, 2016) and organizational outcomes (Ou et al., 2018). Increasingly, scholars and practitioners alike describe humility as a leadership “superpower,” one that tempers ego, encourages learning, and strengthens relationships (Gutman, 2025).

The importance of both trust and humility for leaders and organizations is visible in the values espoused by many of the “Fortune 100 Best Companies to Work For,” where both constructs appear prominently (Dominick et al., 2021).

Despite substantial and growing bodies of research on both trust and humility, these literatures have largely evolved in parallel. Researchers have produced insightful reviews on humility (Nielsen and Marrone, 2018; Luo et al., 2022; Chandler et al., 2023; Kelemen et al., 2023), while others have summarized insights from decades of trust-focused work (Kramer, 1999; Dirks and Ferrin, 2002; Fischer et al., 2020; Dirks and de Jong, 2022; Hancock et al., 2023). Surprisingly, we could find no previous work that systematically integrates both literatures. This omission is surprising given that both trust and humility are fundamental to effective leadership and performance across a variety of settings.

Given the heightened interest in and research on both trust and humility, a comprehensive review of their relationship is needed. Several considerations underscore this need. First, the literature on both constructs spans multiple disciplines, making it difficult to form an integrated view. Second, many researchers define and measure trust and humility differently, leading to conceptual ambiguity. Third, the theoretical and methodological diversity in existing work makes synthesizing existing findings challenging. Finally, the relationship between trust and humility is complex. While numerous studies explore humility as an antecedent to trust, research reveals the relationship between the variables can be more complicated.

Consequently, the purpose of this systematic review is to take stock of and synthesize the growing corpus of scholarly work exploring the relationship between trust and humility. Specifically, this work will address the following research questions:

1. How have trust and humility been conceptualized across various existing studies?
2. What relationships between trust and humility can be identified in these studies?
3. What underlying theories are used to explain the various relationships between trust and humility?
4. Which methodologies are used to study the relationship between trust and humility?

## Theoretical background

### Trust

Researchers have studied trust across various academic disciplines (Dirks and Ferrin, 2002). Consequently, there are at least 129 definitions of trust in the scholarly literature (Fischer et al., 2020). Trust has been viewed as a trait, an emergent state, and as a process (Burke et al., 2007). Further, debate exists over whether trust is a uni- or multi-dimensional construct, with some arguing that trust is more than simply an emotionally-driven psychological state and that it includes both emotional and motivational components (Kramer, 1999) or both affective and cognitive elements (McAllister, 1995). Assessing these various conceptualizations of trust takes many forms, some more reliable and valid than others (McEvily and Tortoriello, 2011).

Much of the existing trust research focuses on interpersonal trust (Hancock et al., 2023), sometimes exploring the concept from the viewpoint of either the trustor or the trustee. Perhaps the most common type of trust studied in organizational research is the trust employees have in their managers or leaders (i.e., a bottom-up perspective), but there are various other studies that explore trust from other perspectives, including whether employees feel that their managers trust them (Zheng et al., 2019).

As mentioned previously, many scholars have focused on trust in a direct leader (e.g., supervisor or manager), while others have focused their research on trust in organizational leadership (e.g., “senior leadership”), with each conceptualization of trust having distinct relationships and outcomes (Dirks and Ferrin, 2002). Still, others have focused on trust in impersonal entities like organizations and institutions (Yang, 2006; Kwantes et al., 2025). Interestingly, Tan and Tan (2000) found that trust in managers or leaders and trust in organizations are distinct constructs, each having its own antecedents and outcomes. Finally, some research explores the concept of trust repair (Kramer and Lewicki, 2010).

Trust has been linked to several positive behavioral and attitudinal outcomes including improved team and individual performance, team learning and knowledge sharing, team and organizational citizenship behaviors, creativity and innovation, turnover, job satisfaction, commitment, and cohesion (Tan and Tan, 2000; Dirks and de Jong, 2022). Because of the noted benefits of trust, a great deal of research explores the antecedent conditions that promote the creation or emergence of trust.

Despite the volume of research focused on trust, there is still much scholars are wrestling with in their efforts to understand the construct. For example, trust is viewed or conceptualized differently, often colored by scholars’ academic background and research focus. In fact, there are at least 129 unique tools used to measure trust (McEvily and Tortoriello, 2011). As a result, questions frequently arise about how to accurately assess trust. For example, McEvily and Tortoriello (2011) question the utility of trust measurement approaches developed in the realm of social psychology for research focused on trust in organizations. Even when a measure purports to assess a specific type of trust, it is not always clear if it does. To wit, there are at least 14 different measures of intraorganizational trust (i.e., between employees and supervisors or among co-workers), but these different measures appear to assess different elements of the trust process (Dietz and Den Hartog, 2006). Further, some contend that all measures purported to assess affect-based trust are flawed and instead assess other aspects of trust—going so far as to suggest that researchers stop using existing measures until something better is developed (Legood et al., 2023). Additionally, Glaeser et al. (2000) note that standard survey questions that purport to assess trust actually appear to measure trustworthiness. Ultimately, many seem to agree that additional work needs to be done not only improving the understanding of trust, but also the tools and methods used to measure it.

### Humility

Like the construct of trust, conceptualizations of humility differ across disciplines, with nuanced definitions and measurement that convolute the literature. For instance, some view humility as a state-like characteristic (Kruse et al., 2014), while others treat it as a stable trait (Nielsen et al., 2010). Research centered on displays of humility or “expressed humility” focuses on actions that demonstrate self-awareness, an appreciation of others, and being teachable (Owens et al., 2013). Similarly, “general humility” is defined as an accurate view of self and the ability to regulate egotism and cultivate an other-oriented stance (Davis et al., 2016). Another type of humility, intellectual humility, refers to an awareness of personal intellectual limitations, although there are differences between psychological and philosophical conceptualizations of the variable (Whitcomb et al., 2017; Porter et al., 2022). Similar to intellectual humility is cognitive humility, a state that is linked to being teachable (Whatley, 2014). Further, some in the medical community have embraced cultural humility (as opposed to cultural competence), defined early-on as a focus on self-evaluation to redress power imbalances in the patient-provider relationship (Tervalon and Murray-Garcia, 1998). However, scholars in fields outside of the medical community more broadly define cultural humility as having an interpersonal, other-oriented stance that is also characterized by respect and lack of superiority toward others due to their cultural background (Hook et al., 2013). Extant research also explores relational humility, which is often defined as an observer’s judgment that someone “(a) is interpersonally other-oriented rather than self-focused, marked by a lack of superiority; and (b) has an accurate view of self – not too inflated or too low” (Hook et al., 2013, p. 226). Researchers have also examined spiritual humility (Davis et al., 2010), political humility (Hodge et al., 2020), and moral humility (Smith and Kouchaki, 2018). Finally, many have investigated a personality trait called Honesty-Humility, which is part of (and assessed by) the HEXACO (Ashton and Lee, 2008). Some contend that Honesty-Humility is related to, but distinct from, actual humility (Owens et al., 2013).

Scholars have noted the need for researchers to address the numerous measurement and methodological concerns for appropriately interpreting and applying empirical findings in the humility literature (Porter et al., 2022). As mentioned above, one measurement issue stems from the numerous conceptualizations of the construct. Even when purporting to assess the same type of humility (e.g., expressed humility), measures may differ (see Chintakananda et al., 2024; Owens et al., 2013). Nonetheless, investigators have assessed humility using several methods, including self-report measures, other-report methods, an implicit association test, and even multi-method approaches (Swain and Murray, 2020). While self-report measures can be easily administered, research has shown a low correlation between one’s self-assessed humility and an assessment of that individual’s humility by an observer (Simpson et al., 2014). Consequently, researchers of humility should consider the measurement source when examining the results of humility studies.

The outcomes purported to result from the various conceptualizations of humility are notable, with impact at the personal, follower, team, and organizational levels. For example, different types of humility have been associated with decreased depression, less stress, and reduced anxiety (Nielsen and Marrone, 2018), as well as increased personal resilience (Chandler et al., 2023). Humility has also been linked to increases in follower self-efficacy (Mao et al., 2019), and research suggests that humble leaders tend to reduce emotional exhaustion among those that work for them (Wang et al., 2018; Yang et al., 2019). Leader humility has also been shown to improve information sharing between people, enhance follower creativity (Wang et al., 2018), and improve job satisfaction and performance (Owens et al., 2013). Even more, there is evidence that humble leaders increase psychological safety (Swain, 2018) and organizational citizenship behaviors among those on their teams (Ding et al., 2020). Humble managers also facilitate innovation (Liu, 2016) and improve overall team performance (Owens and Hekman, 2016). At the organizational level, humble CEOs have been found to create more empowering and ethical climates in their organizations (Kelemen et al., 2023).

While humility has been linked to numerous positive outcomes, researchers have also found evidence of negative effects of humility. For example, Qin et al. (2020) found that leader humility can increase followers’ workplace deviance in some instances. Liu et al. (2024) note that humble leaders may be viewed as lacking decisiveness which can cause followers to question the leader’s effectiveness, especially in time-sensitive situations. Others have found that if followers attribute leader humility to being inauthentic (e.g., an impression management tactic), these followers might perceive their leaders as being hypocritical (Bharanitharan et al., 2019), possibly negatively impacting some of the outcomes traditionally associated with humble managers (Oc et al., 2020). Ultimately, while researchers have learned a great deal about humility, questions about boundary conditions, or the contexts in which positive or negative outcomes associated with humility may manifest, remain unanswered (Wang et al., 2018).

### The relationship between trust and humility

The focus of this paper is the relationship between humility and trust – two increasingly important topics across a variety of leadership related literatures. Numerous research projects suggest humility is an antecedent to trust, with several offering various moderating and mediating variables to further explore the relationship between the two concepts. Still, other works suggest more complex relationships between the two variables. Many of these existing papers leverage different theories in their attempt to explain the relationship between humility and trust. Further, the methods used to explore the complex relationship between trust and humility vary widely and include various qualitative and quantitative approaches. Other papers are purely conceptual. This work will help identify trends and points of note about the research focused on and the relationship between these two important constructs and their importance to leadership.

## Method

The approach employed in this paper incorporates best practices identified for management-focused scholarly reviews (Rojon et al., 2021; Williams et al., 2021). This analysis also follows guidelines set forth in the 2020 Preferred Reporting Items for Systematic Reviews and Meta-Analyses (PRISMA 2020; Page et al., 2021).

The literature review was conducted in October 2024, again in February 2025, and finally in March 2025 using the following databases: APA PsycArticles, APA PsycBooks, and APA PsycInfo via EBSCOhost, as well as JSTOR (limited to the categories of business, management and organizational behavior, labor and employment relations, philosophy, religion, sociology, health, health studies, and psychology), PubMed, the Directory of Open Access Journals, and Semantic Scholar (limited to medicine, psychology, business, sociology, political science, education, and philosophy). The terms “humility AND trust” and “humble AND trust” were used in the searches. A wildcard operator was appended to the front (*trust) and rear (trust*) of the term “trust” to catch variations of the term (e.g., distrust, mistrust, trustworthy, etc.)

In this initial stage, duplicate entries were removed and the remaining works were divided into discrete batches and screened by a single reviewer. Results were retained if they were (1) written in English and (2) initially appeared to address both humility and trust. Full-text versions of the records were requested and excluded if they were not available. After obtaining the full-text versions of the remaining articles, the records were examined to identify additional relevant works cited in those papers.

**Figure 1:**
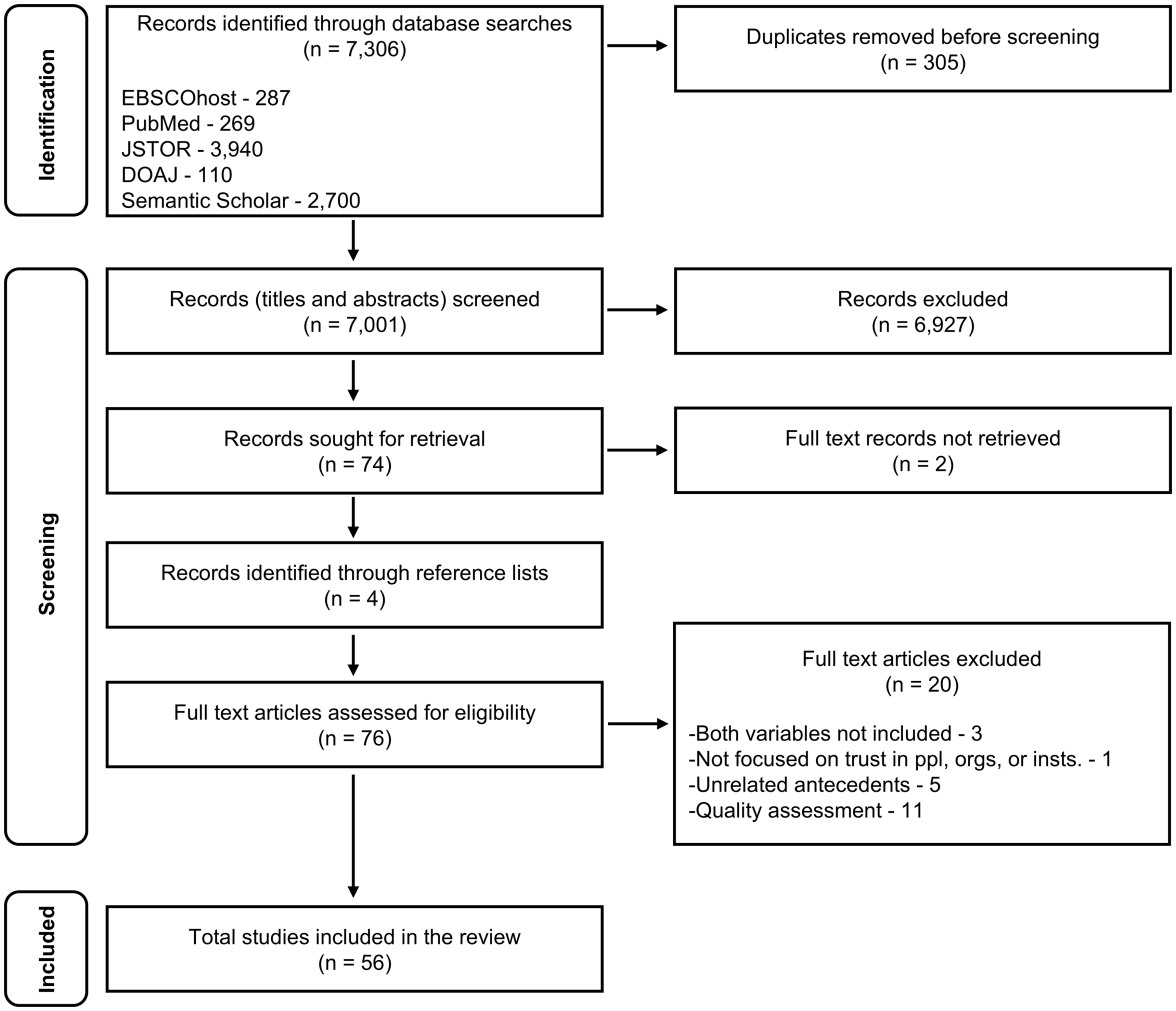
Search strategy based on PRISMA model.

Next, all four researchers independently applied final screening criteria to the full text version of the records on hand. Records were recommended for exclusion if they (1) did not include a focus on both variables (e.g., some papers seemed to examine both concepts, but upon review of the entire paper, they did not); (2) did not focus on trust in people, organizations, or institutions; (3) did not look at the relationship between trust and humility (e.g., both variables were examined in the paper but were unrelated antecedents to some third outcome variable); and 4) did not meet quality standards (e.g., fewer than 10 citations and sample size less than 7). So-called “grey literature” was retained to reduce bias and improve the utility of this systematic review (Adams et al., 2017; Hiebl, 2023). Specifically, book chapters, theses, and dissertations were retained if they explored the relationship between humility and trust and met specific criteria. These “grey literature” sources were identified through the same systematic search procedures as the peer-reviewed articles. Dissertations and theses were considered appropriate for consideration due to their formal academic evaluation processes and often high level of methodological transparency. They were retained if they met the same criteria as journal articles. Book chapters were retained if they provided significant conceptual or theoretical contributions, particularly in areas where empirical research remains limited. Each paper and piece of grey literature that underwent final screening was reviewed independently by all four authors, with each noting whether to retain or reject each item. Reviewers convened and discussed each work on the list collectively to reach a consensus on items to retain or reject. Details of the systematic search are contained in Figure 1.

## Results and findings

This paper explored four research questions. An examination of the literature generated by this systematic review provides some answers to those four questions.

### Conceptualization of trust and humility

Analysis of the relevant literature reveals researchers’ widespread interest in the relationship between humility and trust, not just in the fields of leadership and management, but also in psychology, healthcare, and education focused research. The terms and definitions used for both humility and trust in these studies vary. A complete list of the various types (or at least the terms) used across the literature and the associated definitions are contained in a detailed table contained in the Supplemental materials.

Several papers offer no formal definition of trust (Yeager and Bauer-Wu, 2013; Ito and Bligh, 2016; Rodriguez et al., 2019; Plohl and Musil, 2023; Sijtsma et al., 2023), which is problematic given the different types of trust that exist. Fifty of the fifty-six papers examined did contain a definition of trust, but few of these definitions were the same. An analysis of the definitions revealed a few themes. The most common element identified across the various definitions provided is a willingness to be vulnerable (*n* = 17). There are also themes of emotional connection (*n* = 14) and perceptions of trustworthiness (*n* = 14), both of which emphasize relational elements. A fourth theme identified is one of positive expectations regarding the behavior or intentions of another, which appears in 13 definitions. Finally, an analysis of the data shows that confidence in the competence or reliability of the trustee is also present in 13 definitions, emphasizing the cognitive aspect of trust. We also note that, in some instances, definitions are not consistent across papers even when purporting to examine the same type of trust (e.g., “trust in leader” in Al Hawamdeh, 2023 vs. Basford et al., 2014). Further review found that the most common measure to assess trust in the papers we examined is the tool developed by McAllister (1995) with eight studies using this measure. No more than two of the remaining studies employ a common measure of trust.

In reviewing 49 definitions of humility used in the papers examined, the most frequently mentioned dimension of humility is openness to feedback or new ideas (*n* = 29), which reflects a willingness to remain teachable or embrace feedback. A second prominent theme observed is acknowledging limitations or mistakes (*n* = 17), emphasizing a non-defensive awareness of one’s fallibility. Many definitions included an accurate self-view of one’s strengths and weaknesses (*n* = 15). Other definitions include an appreciation of others (*n* = 9), highlighting a relational orientation toward recognizing the value of other’s contributions. Ultimately, among the papers studied most seem to use a definition similar to the following “an interpersonal characteristic manifested by (a) the willingness to perceive oneself accurately, (b) an appreciation of the strengths and contributions of others, and c) openness to learning new ideas and feedback from others”—the definition of humility originally used by Owens et al. (2013). The second most common type of humility examined in the literature reviewed is intellectual humility. In some studies, intellectual humility is defined similarly to express humility, but in others, it is distinctly different. Recent work has attempted to clarify and refine how intellectual humility is defined and operationalized (Porter et al., 2022).

An analysis of the definitions used in the papers reviewed (when the terms were defined) resulted in the identification of five general categories of humility and five categories of trust. A summary of this synthesis is contained in Table 1. Despite grouping terms, several papers are omitted from Table 1 due to not providing clear definitions or using definitions that varied too greatly from the general categories identified (for both humility and trust). Ultimately, the current literature exhibits substantial heterogeneity in how the two constructs of trust and humility are defined and conceptualized. This variance creates ambiguity regarding whether observed effects reflect the same underlying phenomenon, thereby limiting theoretical precision and cumulative knowledge development.

**Table 1:** Categorization of the types of humility and trust examined in existing research.

Category	Description	Sources
Humility (general)	A multi-dimensional personal virtue comprising primarily internal dispositions including self-awareness, reduced self-focus, openness to growth, and an orientation toward others—many of which are not directly observable. These internal qualities may be expressed behaviorally but are not reducible to observable actions alone; general humility.	Chiu and Hung (2022); Donahue (2018); Exline (2012); Fife and Weeks (2013); Ito and Bligh (2016); Jukanovich (2023); Michalec et al. (2025); Mishra and Mishra (2011); Nielsen et al. (2010); Wang (2019); Wang et al. (2017); Huynh et al. (2020a); Huynh and Dicke-Bohmann (2020); Huynh et al. (2020b); Varma et al. (2024)
Expressed humility	A pattern of outwardly observable behaviors through which people demonstrate accurate self-awareness, openly acknowledge limitations and mistakes, actively seek and accept feedback, highlight and elevate followers’ contributions, and model teachability in interactions with others; expressed humility	Al Hawamdeh (2023); Bharanitharan et al. (2019); Cho et al. (2021); Cojuharenco and Karelaia (2020); Goering et al. (2016); Jia et al. (2024); Liborius and Kiewitz (2022); Liao et al. (2023); Liu (2016); Mertens et al. (2024); Mohamed et al. (2024); Nguyen et al. (2020) O’Kane and Cunningham (2012); Owens and Hekman (2012); Shaw et al. (2025); Simpson et al. (2014); Son and Lee (2023); Son and Yang (2023); Van Tongeren et al. (2023); Wang et al. (2019); Yang et al. (2019); Zhang and Chi (2025)
Intellectual humility	A domain-specific subset of general humility that reflects an individual’s internal recognition of the limits and fallibility of their knowledge, accompanied by openness to revising beliefs and learning from new evidence or perspectives. It represents the cognitive dimension of humility and does not encompass the broader aspects.	Barr et al. (2024); Dormandy (2020); Koetke et al. (2024); Miller (2021); Plohl and Musil (2023); Rodriguez et al. (2019); Tomlinson (2025); Whatley (2016)
Cultural humility	A domain-specific subset of humility that applies to issues of culture, identity, and power. It involves an ongoing process of self-reflection about one’s assumptions those who are different, coupled with a commitment to equitable, respectful engagement across differences.	Dispenza et al. (2021); Hodgin (2014); Yeager and Bauer-Wu (2013)
Honesty-Humility	Personality trait emphasizing moral aspects such as sincerity, fairness, modesty, and low entitlement.	Pfattheicher and Böhm (2018); Ścigała et al. (2020); Sijtsma et al. (2023); Thielmann and Hilbig (2014); Thielmann and Hilbig (2015)
Trust (general)	A psychological state in which an individual is willing to accept vulnerability and uncertainty based on positive expectations about another party’s intentions or behavior.	Cojuharenco and Karelaia (2020); Donahue (2018); Jukanovich (2023); Mishra and Mishra (2011); Pfattheicher and Böhm (2018); Scheuer et al. (2023); Wang (2019)
Affective trust	A form of relationship-based reliance grounded in emotional, where willingness to accept vulnerability arises from perceived care, mutual concern, and interpersonal connection.	Huynh et al. (2020b); Jia et al. (2024); Liborius and Kiewitz (2022); Nguyen et al. (2020); Son and Yang (2023)
Relational/contextual trust	A context-specific form of reliance in which an individual is willing to accept dependence and uncertainty within a defined relationship, role, or system, based on expectations that others will act reliably and in accordance with norms.	Barr et al. (2024); Dispenza et al. (2021); Gazaway et al. (2025); Hodgin (2014); Liu (2016); Mohamed et al. (2024); Miller (2021); Koetke et al. (2024); Wang et al. (2017); Whatley (2016); Zhang and Chi (2025)
Leader trust	A relationship-specific form of trust in hierarchical contexts, defined by a follower’s willingness to be vulnerable to a leader based on expectations of the leader’s competence, benevolence, and integrity.	Al Hawamdeh (2023); Basford et al. (2014); Goering et al. (2016); Liao et al. (2023); Shaw et al., 2025; Son and Lee (2023); Simpson et al. (2014); Yang et al. (2019); Wang et al. (2019)
Trustworthiness	The perceived qualities of an individual or entity (such as competence, integrity, and fairness) that make others willing to accept vulnerability in relation to them.	O’Kane and Cunningham (2012); Thielmann and Hilbig (2014); Thielmann and Hilbig (2015); Van Tongeren et al. (2023); Varma et al. (2024)

In terms of assessment, the most common means to measure humility across the works examined is the Expressed Humility Scale (EHS; *n* = 18; Owens et al., 2013). At least one work purports to examine one type of humility but uses a measure designed to assess another type of humility. Specifically, Basford et al. (2014) claim to examine relational humility, but use the expressed humility scale (EHS) in their work. Relational humility contains an emotional regulation component in its definition that is not present in the EHS. Table 2 contains a listing of the assessment tools used in the papers examined in this study.

**Table 2:** Summary of studies exploring humility and trust.

Source	Field(s) of study	Humility measure	Trust measure	Research design	Measurement source/data	Theories employed
Al Hawamdeh (2023)	Management & Leadership	Adapted from Expressed Humility Scale (EHS); Owens et al. (2013)	Robinson and Rousseau (1994)	Cross-sectional survey	Self-report data	Social Exchange Theory
Barr et al. (2024)	Healthcare	Not reported	Not reported	Conceptual/theoretical analysis	Not reported	Not reported
Basford et al. (2014)	Management & Leadership	Expressed Humility Scale (EHS); Owens et al. (2013)	Podsakoff et al. (1990)	Cross-sectional survey	Self-report data	Transformational Leadership
Bharanitharan et al. (2019)	Management & Leadership	Expressed Humility Scale (EHS); Owens et al. (2013)	Baer et al. (2015)	Multiwave Survey	Multi-source data	Attachment Theory
Chiu and Hung (2022)	Management & Leadership	Humility Scale; Chiu and Hung (2022)	Mayer et al. (1995)	Cross-sectional survey	Multi-source data	Compliance Theory
Cho et al. (2021)	Management & Leadership	Expressed Humility Scale (EHS); Owens et al. (2013)	Mayer and Davis (1999; modified)	Study 1: Experimental, Study 2: Time-lagged survey	Study 1: Self-report data; Study 2: Multi-source data	Social Exchange Theory
Cojuharenco and Karelaia (2020)	Management & Leadership	Expressed Humility Scale (EHS); Owens et al. (2013)	Colquitt et al. (2007)	Study 1: Cross-sectional survey; Study 2–5: Experimental	Study 1: Self-report data; Study 2: Self-report data	Not reported
Dispenza et al. (2021)	Psychology	Cultural Humility Scale (CHS); Hook et al. (2013).	Dyadic Trust Scale; Larzelere and Huston (1980)	Cross-sectional survey	Self-report data	Social Bonds Hypothesis
Donahue (2018)	Management & Leadership	Not reported	Mayer and Gavin (2005)	Mixed-methods	Self-report data + Interview data	Not reported
Dormandy (2020)	Education	Not reported	Not reported	Conceptual/theoretical analysis	Not reported	Not reported
Exline (2012)	Psychology	Dispositional Humility; Bollinger (2010)	Exline (2012); Exline et al. (2012)	Cross-sectional survey	Self-report data	Not reported
Fife and Weeks (2013)	Psychology; Healthcare	Not reported	Trust	Conceptual/theoretical analysis	Not reported	Not reported
Gazaway et al. (2025)	Psychology	Spiritual Humility Scale (SHS); Davis et al. (2010)	Gazaway et al. (2025)	Cross-sectional survey (with dyadic discussion procedure)	Multi-source data	Social Learning Theory; Social Exchange Theory
Goering et al. (2016)	Management & Leadership	Expressed Humility Scale (EHS); Owens et al. (2013)	Mayer and Gavin (2005)	Cross-sectional survey	Multi-source data + Archival data	Not reported
Hodgin (2014)	Education	Not reported	Not reported	Mixed-methods	Self-report + Interview data + Observational data	Not reported
Huynh et al. (2020a)	Healthcare	Not reported	Trust in Physician Scale; Anderson and Dedrick (1990)	Experimental	Self-report data	Not reported
Huynh and Dicke-Bohmann (2020)	Healthcare	Global Humility Subscale (GHS); Davis et al. (2011)	Trust in Physician Scale; Anderson and Dedrick (1990)	Cross-sectional survey	Self-report data	Not reported
Huynh et al. (2020b)	Education; Mgmt & Ldrship	Global Humility Subscale (GHS); Davis et al. (2011)	McAllister (1995)	Cross-sectional survey	Self-report data	Not reported
Ito and Bligh (2016)	Management & Leadership	Not reported	Not reported	Conceptual/theoretical analysis	Not reported	Not reported
Jia et al. (2024)	Management & Leadership	Expressed Humility Scale (EHS); Owens et al. (2013)	McAllister (1995)	Time-lagged survey	Study 1: Self-report data; Study 2: Multi-source data	Social Exchange Perspective
Jukanovich (2023)	Management & Leadership	Not reported	Not reported	Qualitative ethnographic case study	Interview data	Not reported
Koetke et al. (2024)	Healthcare; Psychology	Comprehensive Intellectual Humility Scale (CIHS); Krumrei-Mancuso and Rouse (2016)	Hendriks et al. (2015)	Study 1: Cross-sectional survey; Study 2–5: Experimental	Self-report data	Not reported
Liao et al. (2023)	Management & Leadership	Expressed Humility Scale (EHS); Owens et al. (2013)	Ma and Yao (2012)	Cross-sectional survey	Multi-source data	Not reported
Liborius and Kiewitz (2022)	Management & Leadership	Expressed Humility Scale (EHS); Owens et al. (2013)	McAllister (1995)	Study 1: Longitudinal survey; Study 2: Experimental	Self-report data	Social Exchange Theory
Liu (2016)	Management & Leadership	Expressed Humility Scale (EHS); Owens et al. (2013)	Gao et al. (2011)	Cross-sectional survey	Self-report data	Social Exchange Theory
Mertens et al. (2024)	Healthcare	Expressed Humility Scale (EHS); Owens et al. (2013)	Yang and Mossholder (2010)	Cross-sectional survey	Self-report data	Not reported
Michalec et al. (2025)	Healthcare	Tangney (2000); Gruppen (2015)	Not reported	Qualitative interview study	Interview data	Not reported
Miller (2021)	Legal	Not reported	Not reported	Conceptual/theoretical analysis	Not reported	Not reported
Mishra and Mishra (2011)	Management & Leadership	Not reported	Not reported	Conceptual/theoretical analysis	Not reported	Positive Organizational Scholarship
Mohamed et al. (2024)	Healthcare	Expressed Humility Scale (EHS); Owens et al. (2013)	Dirks and Ferrin (2002)	Cross-sectional survey	Self-report data	Not reported
Nguyen et al. (2020)	Management & Leadership	Expressed Humility Scale (EHS); Owens et al. (2013)	McAllister (1995); Kim et al. (2016)	Time-lagged survey	Self-report data	Social Exchange Theory
Nielsen et al. (2010)	Management & Leadership	Not reported	Not reported	Conceptual/theoretical analysis	Not reported	Not reported
O’Kane and Cunningham (2012)	Management & Leadership	Not reported	Not reported	Qualitative case study	Interview data + Archival data	Not reported
Owens and Hekman (2012)	Management & Leadership	Not reported	Not reported	Qualitative interview study	Interview data	Not reported
Pfattheicher and Böhm (2018)	Psychology	HEXACO-60; Ashton and Lee (2009)	Berg et al. (1995); Kreps (1990)	Study 1: Cross-sectional survey; Study 2–5: Experimental	Study 1: Self-report data; Study 2–5: Behavioral data + Self-report data	Not reported
Plohl and Musil (2023)	Psychology	Comprehensive Intellectual Humility Scale (CIHS); Krumrei-Mancuso and Rouse (2016)	Nadelson et al. (2014)	Cross-sectional survey	Self-report data	Not reported
Rodriguez et al. (2019)	Psychology	Adapted Cultural Humility Scale (Hook et al., 2013)	Rodriguez et al. (2019)	Cross-sectional survey (with dyadic discussion procedure)	Multi-source data	Not reported
Scheuer et al. (2023)	Management & Leadership	Not reported	Not reported	Qualitative interview study	Interview data	Social Exchange Theory; Knowledge Transfer
Ścigała et al. (2020).	Psychology	HEXACO-60; Ashton and Lee (2009)	Lying-Trust Game; Ścigała et al. (2020)	Experimental	Behavioral data + Self-report data	Not reported
Shaw et al. (2025)	Management & Leadership	Expressed Humility Scale (EHS); Owens et al. (2013)	Ma and Yao (2012)	Time-lagged survey	Self-report data	Social Identity Theory
Sijtsma et al. (2023)	Psychology	Brief HEXACO Inventory (BHI); De Vries (2013)	Trust Game; Sijtsma et al. (2023)	Experimental	Behavioral data + Self-report data	Not reported
Simpson et al. (2014)	Management & Leadership	Simpson et al. (2014) w/ questions from Dennis and Bocarnea (2005); Park et al. (2004)	Rego and Cunha (2008); Dennis and Bocarnea (2005); Simpson et al. (2014)	Cross-sectional survey	Multi-source data	Not reported
Son and Lee (2023)	Management & Leadership	Expressed Humility Scale (EHS); Owens et al. (2013)	McAllister (1995)	Cross-sectional survey	Self-report data	Attachment Theory
Son and Yang (2023)	Management & Leadership	Expressed Humility Scale (EHS); Owens et al. (2013)	McAllister (1995)	Cross-sectional survey	Self-report data	Social Information Processing Theory
Thielmann and Hilbig (2014)	Psychology	German HEXACO-60 (Moshagen et al., 2014) from HEXACO-60; Ashton and Lee, 2009	McEvily et al. (2012)	Cross-sectional survey	Behavioral data + Self-report data	Social Projection Theory
Thielmann and Hilbig (2015)	Psychology	German HEXACO-60 (Moshagen et al., 2014) from HEXACO-60; Ashton and Lee, 2009	Berg et al. (1995)	Cross-sectional survey	Behavioral data + Self-report data	Not reported
Tomlinson (2025)	Management & Leadership	Not reported	Not reported	Conceptual/theoretical analysis	Not reported	Not reported
Van Tongeren et al. (2023)	Management & Leadership	Not reported	Van Tongeren et al. (2023)	Experimental	Self-report data	Trust Signaling Hypothesis
Varma et al. (2024)	Psychology	Humility Semantic Differential; Rowatt et al. (2006)	Huang and Murnighan (2010)	Study 1: Experimental; Study 2: Cross-sectional survey	Self-report data	Self Categorization Theory
Wang (2019)	Psychology	General Humility Scale; Hill et al. (2015) and Relational Humility Scale; Davis et al. (2011)	Rempel et al. (1985).	Cross-sectional survey	Multi-source data	Not reported
Wang et al. (2017)	Management & Leadership	General Humility Scale; Hill et al. (2015) and Relational Humility Scale; Davis et al. (2011)	Rempel et al. (1985)	Cross-sectional survey	Multi-source data	Not reported
Wang et al. (2019)	Management & Leadership	Expressed Humility Scale (EHS); Owens et al. (2013)	McAllister (1995)	Time-lagged survey	Multi-source data	Social Exchange Theory; LMX Theory
Whatley (2016)	Psychology	Healthy Humility Index (HHI); Quiros (2006)	Kirkman et al. (2006)	Cross-sectional survey	Self-report data	Not reported
Yang et al. (2019)	Management & Leadership	Expressed Humility Scale (EHS); Owens et al. (2013)	Jarvenpaa and Leidner (1999)	Time-lagged survey	Multi-source data	Rational Choice Theory
Yeager and Bauer-Wu (2013)	Healthcare	Not reported	Not reported	Conceptual/theoretical analysis	Not reported	Not reported
Zhang and Chi (2025)	Education	Expressed Humility Scale (EHS); Owens et al. (2013)	Zhang and Chi (2025)	Time-lagged survey	Self-report data	Social Exchange Theory; Self-Determination Theory

### Relationship between trust and humility

**Figure 2:**
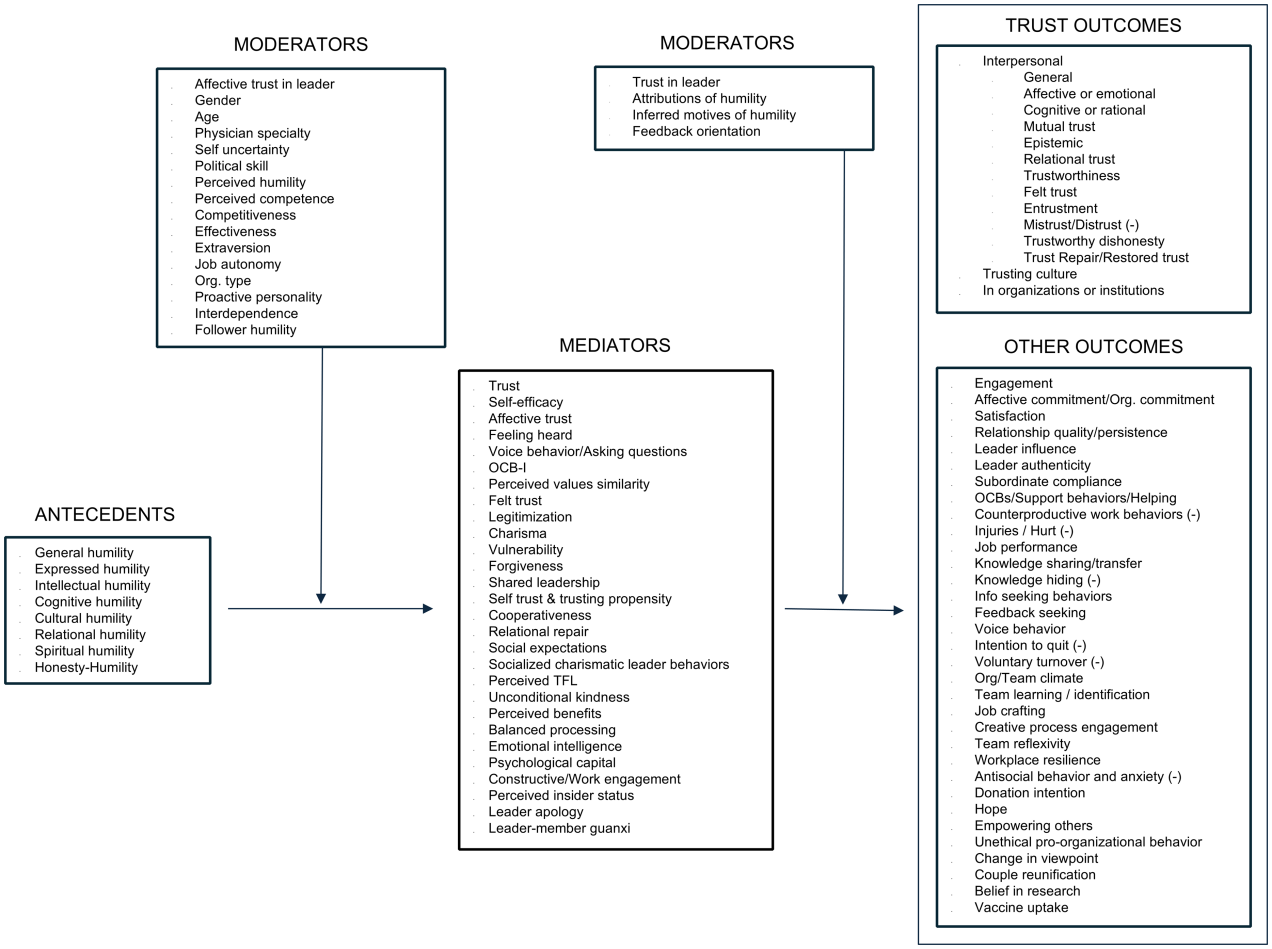
Summary of relationship between humility and trust.

A review of the existing literature suggests that the relationship between trust and humility is primarily one where the exhibition of humility leads to the development of trust (see Figure 2). The majority of studies examined humility as an antecedent to trust where trust is the outcome variable of interest. However, several papers note other relationships where trust mediates (Nguyen et al., 2020) or moderates (Son and Yang, 2023) the relationship between humility and some other outcome variable of importance to leadership. Other work contained in our review postulates a mutually reinforcing relationship between trust and humility (Jukanovich, 2023). Still other research finds unique relationships between the two variables, like Chiu and Hung (2022) who found that humility acts as a moderator in a model that links trustworthiness and compliance.

Focusing on humility as an antecedent to trust, results are inconsistent. For example, while some found Honesty-Humility to be related to trust (Thielmann and Hilbig, 2015; Pfattheicher and Böhm, 2018), others found no relationship, at least in adolescent populations (Sijtsma et al., 2023). Developmental moderators such as age appear to help explain these differences (Li et al., 2025). Additionally, intellectual humility has been shown to lead to trust (Dormandy, 2020; Koetke et al., 2024), but not in all instances. In some cases, it appears the other’s perception of a partner’s intellectual humility is key to feelings of trust (Rodriguez et al., 2019), while only openness to revising one’s viewpoint (one aspect of intellectual humility) predicted trust in science (Plohl and Musil, 2023). See Table 3 for a summary of the hypothesized relationships between humility and trust.

**Table 3:** Relationships linking humility and trust.

Source	Antecedent(s)	Moderator(s)	Mediator(s)	Outcome(s)
Al Hawamdeh (2023)	Humble leadership		Trust in leader Self-efficacy	Knowledge hiding (−)
Barr et al. (2024)	Intellectual humility		Expressed trust	Vaccine uptake
Basford et al. (2014)	Source credibility		Leader apology, Perceived humility, Perceived TFL Forgiveness	Trust in leader Satisfaction with supervision LMX relationship quality Org. commitment
Bharanitharan et al. (2019)	Leader humility		Follower feeling trusted (felt trust) Follower self-efficacy for voice	Follower challenging voice Follower defensive voice
Chiu and Hung (2022)	Trustworthiness Authority	Leader humility		Subordinate compliance
Cho et al. (2021)	Humble leadership	Job autonomy	Employee feeling trusted	Job performance OCB toward co-workers OCB toward the organization
Cojuharenco and Karelaia (2020)	Asking questions	Prior doubt in competence Prior doubt in humility	Perceived competence Perceived humility	Trust Helping intentions
Dispenza et al. (2021)	Perceptions of cultural humility Collective self-esteem Attachment styles			Perceptions of relationship quality (e.g., dyadic trust, adjustment, and relationship commitment)
Donahue (2018)	Consistency Humility		Trust	Leader authenticity
Dormandy (2020)	Intellectual humility			Epistemic trust in self and others
Exline (2012)	Humility			Pos. emotional response (e.g., gratitude) Neg. emotional responses (e.g., shame, mistrust) (−)
Fife and Weeks (2013)	Humility Empathy Commitment Apology		Forgiveness	Couple reunification Restored trust
Gazaway et al. (2025)	Spiritual humility	Actor-partner interdependence		Trust Partner’s self-report hurt (−) Change in viewpoint on religious issues
Goering et al. (2016)	Leader humility	Follower humility	Trust in leader	OCB Counterproductive work behaviors (−) Injuries (−)
Hodgin (2014)	Cultural humility			Trusting relationships
Huynh et al. (2020a)	Physician humility	Physician gender Physician speciality General effectiveness		Trust Patient out-of-pocket expenditure Patient satisfaction Patient likelihood to recommend physician Adherence intentions
Huynh and Dicke-Bohmann (2020)	Clinician humility			Trust Patient satisfaction Self-report health
Huynh et al. (2020b)	Coach humility		Affect-based trust Cognition-based trust (null finding)	Player perception of coach influence Player perception team climate
Ito and Bligh (2016)	Humility Self-awareness Courage to acknowledge imperfections		Vulnerability, Charisma	Trust Psychological safety Egalitarian leader-follower relationship
Jia et al. (2024)	Leader humility		Follower affective trust Follower self-efficacy Follower perceived insider status	Knowledge exchange Helping Voice
Jukanovich (2023)	Leader humility Leader trustworthiness			Perceived trustworthiness Perceived humility
Koetke et al. (2024)	Intellectual humility	Gender of humble scientist (null finding) Race/ethnicity of humble scientist (null finding)		Trust in scientists Belief in scientists’ research Information seeking behavior
Liao et al. (2023)	Humble leadership	Feedback orientation	Supervisor trust	Feedback-seeking behavior
Liborius and Kiewitz (2022)	Leader humility	Follower’s competitiveness	Affective trust	Intention to quit (−) Voluntary turnover (−)
Liu (2016)	Humble leader behavior		Trust in leadership Positive affect	Employee’s voice behavior
Mertens et al. (2024)	Leader humility Psychological safety climate		Affective trust Feeling heard Voice behavior	Affective commitment
Michalec et al. (2025)	Humility			Trust (in training) Pivot and adapt to new information
Miller (2021)	Intellectual humility			Trust
Mishra and Mishra (2011)	Humility Courage Authenticity		Self-trust and trusting propensity, Demonstrating trustworthiness, Trusting others	Creating and sustaining hope Empowering others
Mohamed et al. (2024)	Humble leadership			Workplace resilience Organizational trust
Nguyen et al. (2020)	Leader humility		Affective trust, Work engagement, OCB-I	Knowledge sharing intention
Nielsen et al. (2010)	Leader humility	Follower attributions of humility	Socialized charismatic leader behaviors	Identification with leader Trust in leader Self-efficacy Motivation Willingness to sacrifice
O’Kane and Cunningham (2012)	Leadership humility			Trust
Owens and Hekman (2012)	Humble behavior	Perceptions of leader competence Perceptions of leader sincerity Threat and time pressure Learning culture Hierarchical adherence	Legitimization of followers’ developmental journeys Legitimization of uncertainty	Trust Fluidity of organizing Continuous small change Follower engagement Psychological freedom
Pfattheicher and Böhm (2018)	Honesty-Humility	Self-uncertainty	Social expectations	Trust
Plohl and Musil (2023)	Intellectual humility			Trust in science (null finding)
Rodriguez et al. (2019)	Intellectual humility			Trust (null finding)
Scheuer et al. (2023)	Humility Competence	Age diversity	Workers’ perceived benefits of knowledge transfer Perceived trust within dyad	Knowledge transfer
Ścigała et al. (2020)	Honesty-Humility	Trustworthiness		Trustworthy dishonesty
Shaw et al. (2025)	Leader humility	Leader trust	Perceived insider status Leader-member guanxi	Unethical pro-organizational behavior
Sijtsma et al. (2023)	Honesty–humility Agreeableness Conscientiousness	Age		Trust behavior (null finding)
Simpson et al. (2014)	Perceived leader humility	Leader immodesty	Balanced processing	Trust in leader
Son and Lee (2023)	Leader humility	Gender Leader trust		Job crafting
Son and Yang (2023)	Leader humility	Leader trust (affect-based)	Relation-oriented shared leadership	Team reflexivity
Thielmann and Hilbig (2014)	Honesty-Humility		Cooperativeness Entitlement (null finding)	Trustworthiness expectations
Thielmann and Hilbig (2015)	Honesty-Humility		Unconditional kindness Positive reciprocity (null finding) Negative reciprocity (null finding)	Trustworthiness
Tomlinson (2025)	Affective (political) polarization	Trust repair through constructive engagement (Intellectual humility, Listening, Civility, Focus on agreement)		Antisocial behavior (−) Anxiety (−) Mutual trust Relationship persistence
Van Tongeren et al. (2023)	Leader humility Leader competence	Gender Organization type	Trustworthiness, Trust	Leader ratings Donation intentions
Varma et al. (2024)	Country of origin Job Humility		Perceived trustworthiness Perceived values similarity	Social–emotional support Instrumental support
Wang (2019)	Humility	Gender	Emotional intelligence	Trust Trust repair
Wang et al. (2017)	Humility		Relational repair	Trust
Wang et al. (2019)	Leader Humility	Political skill	Interpersonal justice Trust in supervisor	Counterproductive work behaviors (−)
Whatley (2016)	Cognitive humility		Psychological capital	Team trust Team identification Team learning
Yang et al. (2019)	Leader-perceived capability of followers	Inferred motives of leader expressed humility	Leader expressed humility	Follower trust in leader
Yeager and Bauer-Wu (2013)	Mindfulness Reflection		Cultural humility	Honesty Trustworthiness
Zhang and Chi (2025)	Humble teacher leadership	Proactive personality	Student trust, Psychological empowerment	Student creative process engagement

*Mediators and moderators separated by a comma are serial; those listed on separate lines occur in parallel.

### Underlying explanatory theories

Researchers have provided a few theoretical explanations for the humility-trust relationship. Of note, however, authors of fewer than half of the papers in this review used an existing theory to explore or explain the relationship between trust and humility. The most common theory employed was Social Exchange Theory (SET; *n* = 10). SET posits that relationships develop through reciprocal exchanges that foster mutual trust and obligation (Cropanzano and Mitchell, 2005). Nearly all the studies employing SET were in the field of management and leadership and focused on relational or leader-based humility, and in all of these papers, humility was explored as an antecedent, with trust as a mediator for other behaviors. In the studies referencing SET, humble leaders appear to engender reciprocity with team members, which leads to follow-on positive behaviors, such as job commitment (Liborius and Kiewitz, 2022), organizational citizenship behaviors (Cho et al., 2021), or knowledge sharing (Nguyen et al., 2020). The other most applied theory in the body of work we examined is Attachment Theory (*n* = 2). Attachment Theory highlights emotional bonds and security (Bharanitharan et al., 2019; Son and Lee, 2023), which humble leaders appear to engender in and provide to their followers. A listing of the various theories employed in the existing body of research are in Table 2.

An analysis of the theoretical explanations employed across the literature suggests that Social Exchange Theory (SET) offers the most common baseline account for the relationship between humility and trust. Specifically, SET explains how humble behavior elicits reciprocal responses from others, thereby fostering a willingness to accept vulnerability. This framework is particularly well suited to explaining the cognitive dimension of trust, as it emphasizes rational assessments of exchange relationships and expectations of future behavior. However, reliance on SET assumes relatively uniform effects across individuals and contexts, offering limited explanatory power for observed variability in outcomes. In contrast, Leader–Member Exchange (LMX) theory shows promise in accounting for such variation by focusing on the quality of dyadic relationships within hierarchical settings. At the same time, Attachment Theory provides a complementary lens through which to view the relationship by explaining how humility may foster affective or emotional forms of trust- often with various mediators helping explain the relationship. Finally, research examining trustworthiness may benefit from the application of Attribution Theory, as perceptions of a leader’s humility may be interpreted through attributional processes that shape judgments of competence, integrity, and benevolence—which impact perceptions of trust (Tomlinson, 2018). Ultimately, due to the varied conceptualizations of the trust and humility constructs, a multi-theoretical approach is necessary to fully capture the complex and multidimensional relationship between humility and trust.

### Methodological approaches of study

Our analysis shows that the relationship between humility and trust has been studied using a variety of methodological approaches. Research design and data type are important factors to consider when examining study results, especially in instances where results are inconsistent (as they are in some instances in the humility/trust research). Empirical studies included in the review range from qualitative case study examinations to quantitative, experimental approaches conducted in a lab setting. Some of the papers included in the review are purely conceptual yet they yield insights for both researchers and practitioners. No trend appears to exist for the choice of method and the field of study, with scholars in healthcare, management, education, and psychology employing conceptual, qualitative, and quantitative approaches in their research of humility and trust. However, those papers that are conceptual or qualitative appear to be less specific in the type of trust they are studying. The same does not appear to hold for humility, with these conceptual and qualitative papers clearly specifying the type of humility they are exploring. See Table 2 for a useful summary of research design and data types/sources used in these studies.

## Concluding discussion

This systematic review addressed four research questions focused on the relationship between the constructs of trust and humility—their conceptualizations, the nature of their relationship, the theories used to explain those relationships, and the methodological approaches employed in their study. Collectively, the work examined in this paper reveals that while interest in the intersection of trust and humility is growing across leadership-related fields, the research remains fragmented, with room for improvement and additional contributions. Nevertheless, important patterns and insights emerged from this examination.

First, despite the mature body of research on both subjects, both trust and humility are defined inconsistently across the literature examined. For humility, the most frequently used conceptualization reflects the behavioral manifestation of humility (most often from leaders/those in power)—mirroring Owens et al. (2013) expressed humility. However, an increasing number of studies are examining other aspects of humility, like intellectual humility and cultural humility. For trust, many works offer no clear definition at all. This lack of conceptual clarity for both elements remains a barrier and hinders cross study comparison, theory building, and practical application. Moving forward, researchers should explicitly define which type of trust and humility they are studying and ensure measurement tools align with those definitions. For those interested in leadership and organizational performance, we recommend examining expressed humility. The definition of Expressed Humility is similar to or encompasses many of the other variations of humility (e.g., Expressed Humility is very similar to General Humility, while Intellectual Humility is viewed as a sub domain of General Humility and includes an understanding of one’s limitations—which is captured in Expressed Humility. Further, the other-report EHS is a valid, reliable measure for assessment that avoids the social desirability bias present in many of the self-report measures of humility. That said, it should be noted that informant-report (also referred to as observer- or other-report) can also suffer from systemic biases (McCrae and Weiss, 2007), observational limitations (i.e., not having access to private thoughts; Olino and Klein, 2015), or even manipulation (Walker and MacCann, 2024). Additionally, while primarily used in research focused on medical professionals’ interactions with patients, cultural humility could yield insights for leaders in other organizational settings and their relationships with followers or subordinates. The definition of Cultural Humility employed by Hook et al. (2013) has wider application and would seem to offer more promise for both practitioners and researchers interested in the topic, although most existing measures of Cultural Humility appear to be focused on a clinical setting and may need to be adjusted for broader organizational use. Regardless of the type of humility examined, researchers should consider employing multimethod approaches (e.g., combination of self-report, other-report, and/or behavioral measures) in their assessments, when feasible, to improve validity (McCrae and Weiss, 2007; McDonald, 2008).

Regarding trust, we recommend the use of McAllister’s (1995) definition of trust, which includes a focus on affective and cognitive elements. However, while McAllister (1995) assessment scale is a validated and widely adopted attitudinal measure, future work should employ methods to gauge behavioral trust. With that in mind, trust games are a useful (and practical) way to assess behavioral trust in a laboratory environment, but they do come with their own weaknesses that should not be ignored (Alós-Ferrer and Farolfi, 2019). Employing qualitative methods and observing trust in actual teams and organizations may be the best way to assess the construct (outside of using an fMRI which is not practical for many researchers).

Second, the dominant pattern across the literature examined positions humility—particularly leader humility—as an antecedent to trust, which often then mediates some other downstream outcome (e.g., knowledge sharing, OCBs, etc.). However, this relationship is not universal. In instances where trust is a mediating mechanism in some more complex relationship, affective trust is often a central mediator, suggesting emotional bonds are key in humility-trust dynamics. It also appears that gender and organizational context frequently moderate the relationship between humility and trust, indicating the importance of situational factors in the interaction. Some of the inconsistent results noted in the trust-humility relationship reinforce the importance of factoring in context or boundary conditions when studying the relationship between the two constructs. These inconsistencies also suggest the need for further research.

Further, fewer than half of the reviewed studies employed a formal theoretical framework in their work, with Social Exchange Theory (SET) appearing most often—particularly in leadership and management research. In teams and organizations, the SET framework can explain why humble behavior encourages others to invest trust—and why trust, once established, may reinforce humility (an area noted for further work). While SET offers a useful framework for understanding the interaction between trust and humility, it is an incomplete account—largely focusing on relational reciprocity, which relies on the cognitive aspect of trust. Trust and humility are affective and identity-laden constructs, suggesting that further work exploring attachment theory may offer richer or complementary explanatory power. Further, trust often occurs between those of varying levels of power—suggesting Leader Member Exchange (LMX) could provide additional explanatory insights, and perhaps help explain differences in study outcomes.

Regarding the methodological approaches employed in studying both trust and humility, experimental designs that manipulate humility (e.g., via vignettes or interventions) remain rare, weakening arguments about causality. Conceptual papers tend to treat trust generically, whereas empirical studies more often specify the type of trust being studied, but often employ a survey approach to assessing trust, which does not always accurately reflect actual trust (or lack thereof). Measurement-misalignment is also a concern, with researchers using incorrect tools to assess both trust and humility at times. To advance the field, greater rigor in construct-operationalization alignment and increased use of experimental and longitudinal designs is needed. Using validated scales like the EHS will help, but a reliable, valid method for assessing cultural humility outside of a purely clinical environment is needed, as are behavioral means to assess trust related variables. When feasible, researchers should consider employing multimethod approaches in their assessments.

Additionally, qualitative work could help identify additional explanatory theories or narrow down which mediators or moderators influence the relationship between trust and humility, especially in instances where humility is examined as an antecedent to trust. Specifically, ethnomethodology and conversation analysis may provide insights. By focusing on observable conversational practices and responses, researchers might better understand the mechanisms linking humility to trust, moving beyond weaknesses in many current studies (e.g., self-report data or capturing attitudinal rather behavioral outcomes of trust).

### Contribution to theory and practice

Work exploring the link between humility and trust continues to be a popular topic (several papers exploring the link between the two topics have emerged since this paper was submitted for publication). Humility work appears to be fragmenting—with researchers exploring various types or domain-specific aspects of humility (e.g., expanded focus on intellectual humility and growing interest in cultural humility), while focus on trust appears to be converging to emphasize its affective/emotional component (especially as a mediator). Noted inconsistencies in outcomes across studies can come from examining different types of humility and trust. It is imperative to be clear in operationalizing terms and to intentionally design studies. As noted, moderators and context matter, but these factors do not alone account for the variance in outcomes noted. Over generalization is likely an issue as a result of a lack of clarity in conceptualization. Interested researchers could focus on replicating existing studies to address some of these issues.

### Limitations

A weakness of this systematic review was the use of a single reviewer for title and abstract screening during the initial phase of study. This approach may have introduced selection bias and limit reproducibility compared to the use of dual independent screening. That said, methodological guidance for systematic reviews recognizes the need to balance comprehensiveness with feasibility (e.g., resource constraints; Hiebl, 2023). In particular, large-scale reviews like this study frequently require trade-offs to ensure the review process remains manageable, especially during early identification and screening phases when thousands of records may be retrieved (Rojon et al., 2021). To mitigate this limitation, all studies that passed title and abstract screening underwent full-text review by multiple authors, introducing a second level of scrutiny at the stage most critical to final inclusion decisions.

Another weakness of this systematic review is the omission of non-English papers. The decision to exclude non-English-language publications reflects a common practical constraint but may lead to language or publication bias, whereby findings from non-English-speaking contexts are underrepresented. Given that both trust and humility are constructs with potential cultural variability, this exclusion could skew the evidence base toward Western perspectives. While several papers in this review include sample populations from a variety of countries and cultures, future work could enhance robustness by incorporating multilingual search strategies and formal assessments of publication bias (e.g., funnel plot analyses or statistical tests). There is also the possibility that we did not find all relevant papers by limiting our search to the databases we employed in our search, although we believe we found and included the majority (and most significant) articles addressing the topic at hand.

Another potential weakness involves the exclusion of work focused on Servant Leadership (SL), which often suggests a tie between that theory and the outcome of trust (Joseph and Winston, 2005; Sendjaya and Pekerti, 2010). Papers exploring SL theory were not included because the studies examined did not disentangle humility from the other elements comprising SL. To elaborate, servant leaders *are* humble, but a humble leader is not automatically a servant leader, as humility alone does not guarantee the active, people-first behaviors indicative of servant leaders. Excluding SL from this paper’s search aligns with findings by other researchers—who acknowledge that while overlap / similarity exists between humility and different moral-oriented leadership theories (i.e., servant leadership, ethical leadership, authentic leadership), the concepts are conceptually distinct and should not be combined (see Lemoine et al., 2019; Owens and Hekman, 2012; Rego et al., 2017; Van Dierendonck, 2011).

### Directions for future research

In addition to the work to address the noted limitations in our study, there are several shortfalls and unanswered questions in the existing corpus which future research should address. First, future work should include a meta-analysis of quantitative studies—once the number of these types of studies increases. This meta analysis could examine the strength of the relationship between trust and humility—possibly focusing on specific types of humility and resulting specific types of trust.

As noted in the concluding discussion, future work can also help address some of the noted inconsistencies in the humility-trust relationship. Specifically, future work could focus on exploring boundary conditions. For example, Xu et al. (2022) suggest that humble leaders are not perceived to be as effective during times of uncertainty, possibly because trust in them suffers. However, work by Kolditz (2010) suggests that humble leaders in dangerous contexts actually increase trust, while Michalec et al. (2025) suggest humility builds trust in oneself during times of crisis or uncertainty. Consequently, additional work exploring the relationship between humility and trust in times of uncertainty or crisis is warranted.

Similarly, with the increase in hybrid and remote work, research is needed that explores the impact of humility on trust in virtual environments. While there is evidence that humility can be perceived in virtual interactions (Swain, 2018), it is unclear if humility has the same effect on facilitating trust in on-line settings as it does in face-to-face interactions.

As noted by Dirks and de Jong (2022), the speed at which trust develops is a largely unexplored area. Future work could explore if and how humility impacts the speed at which trust develops compared to other antecedents. Given that humility has been linked to trust repair (Tomlinson, 2025), future work could also explore the impact of humility on the robustness or the maintenance of trust between leaders and those they lead. It is possible that the trust that develops from humble leaders is stronger or more enduring than trust that develops from other types of leader; the same may be said of the trust leaders have in subordinates who display humility.

Finally, the proposition that trust influences humility warrants further empirical investigation. Jukanovich (2023) observes that perceived humility contributes to trust—something widely supported in the leadership literature—but she also suggests a reciprocal effect, whereby trust enhances positive perceptions of a leader’s humility. Prior research demonstrates that humility and gratitude mutually reinforce one another (Kruse et al., 2014), and that trust and healthy relationships likewise operate within a virtuous cycle (Knowledge at Wharton, 2010). By extension, a similar reciprocal dynamic may exist between trust and humility. Future experimental research should examine whether such a relationship exists not only in terms of perceived humility, but also in relation to leaders’ actual behaviorally grounded humility.

## Data Availability

The original contributions presented in the study are included in the article/Supplementary material, further inquiries can be directed to the corresponding author.
